# Breathwork for Chronic Stress and Mental Health: Does Choosing a Specific Technique Matter?

**DOI:** 10.3390/medsci13030127

**Published:** 2025-08-13

**Authors:** Adam Siebieszuk, Adam Filip Płoński, Marcin Baranowski

**Affiliations:** 1 Department of Physiology, Medical University of Białystok, Mickiewicza 2c, 15-222 Białystok, Poland; 2 Department of Gastroenterology and Internal Medicine, Medical University of Białystok, M. Skłodowskiej-Curie 24a, 15-276 Białystok, Poland

**Keywords:** pranayama, HRV, slow deep breathing, vagus nerve, parasympathetic, breathing exercises, diaphragm, respiration

## Abstract

Modern society faces a growing prevalence of mental disorders, with stress emerging as a critical factor affecting mental well-being. In recent years, breathwork has gained public and scientific recognition as a promising approach for enhancing psychological health. Despite the rapid growth in research, the field remains fragmented due to the diversity of breathing techniques. Moreover, recent findings have challenged several foundational concepts traditionally believed to underlie the therapeutic effects of breathwork. This review offers a comprehensive overview and comparison of the most widely practiced breathing techniques, with a focus on addressing key theoretical issues. We examine the primary psychophysiological pathways and mechanisms of breathwork, highlighting its influence on the nervous system as central to its effectiveness. We critically evaluate the role of breathing variables, including pace, ratio, breathing route, attention, and the use of biofeedback, in promoting the long-term neurobiological changes that have been associated with improved mental health. We argue that most breathwork techniques share core neurophysiological mechanisms that benefit well-being, regardless of the theoretical differences between specific techniques. Accumulating evidence suggests breathwork may serve as both a preventive and adjunctive therapy for chronic stress, anxiety, and depression, given its potential to target key risk factors and produce clinically relevant outcomes. Contemporary breathwork research, however, is limited by inconsistent study quality and methodological heterogeneity. By synthesizing current evidence and identifying critical knowledge gaps, this review aims to guide future research and advance understanding of breathwork’s therapeutic potential.

## 1. Introduction

Ventilation is generally an automatic process controlled by the respiratory center in the brainstem, primarily responsible for gas exchange [1]. Beyond its physiological function, breathing is closely linked to emotions and psychological states [2,3,4]. Respiratory rate increases under heightened cognitive load [5], as well as during mental stress [6,7]. Furthermore, the way a person breathes has been shown to provide valuable insights into one’s fitness level and general health [8,9]. Changes in breathing patterns may indicate pain and serve as early signs of deteriorating health in various medical conditions [7,10,11].

What makes breathing unique among other vital functions is humans’ ability to voluntarily control it for various health, performance, and psychological benefits [12,13]. This distinctive characteristic of breathing has fascinated humanity since ancient times [14,15,16]. Among early civilizations, Chinese culture emphasized the importance of proper breathing, while in India, pranayama, the practice of conscious breath control, was being developed [13,17,18].

Today, the diverse range of breathing techniques, encompassing the intentional regulation of respiration biomechanics and parameters, is collectively known as “breathwork” [16,19]. Recently, breathwork has gained widespread public recognition and growing interest in academic research, reflecting a rising trend of people actively seeking innovative ways to alleviate stress and enhance their mental health [20,21,22].

Previous research has indicated breathwork’s promising role in promoting overall mental well-being [13,19,23,24]. However, despite numerous studies, many aspects and mechanisms associated with its positive effects remain unexplored. One of the most important questions is which specific parameters and their associated mechanisms yield the most pronounced effects on physiological outcomes. Given the vast diversity of breathwork techniques—which vary in breathing pace, pattern, pathway, attentional focus, and modality usage—identifying the key factors driving their effectiveness is crucial for proper standardization and broader clinical application.

This review explores the potential of voluntary breath regulation as a preventive and therapeutic strategy for addressing mental health problems. We highlight the detrimental influence of allostatic load, discussing how breathwork may regulate psychophysiological responses to stress and improve psychological well-being. The article provides an in-depth analysis of the pathways and physiological processes through which breathwork impacts human health, focusing on its effects on the nervous and cardiovascular systems. By examining the shared foundations underlying the positive outcomes of different techniques, we aim to uncover whether specific breathwork techniques or the broader act of regulating breathing holds greater significance in achieving these benefits. Finally, we discuss the role of breathwork in clinical practice.

## 2. Search Strategy and Selection Criteria

The literature search was conducted using the Scopus, Web of Science, and PubMed databases, covering all articles published up to and including January 2025. The search was restricted to full-text articles published in English that were relevant to the scope of this review, with priority given based on their level of evidence, with particular emphasis placed on studies published within the last decade. Additional references were identified by reviewing the reference sections of relevant publications by leading experts in the field. The search included the following terms, along with their variants and combinations: “breathwork,” “breathing,” “pranayama,” “slow,” “deep,” “resonant,” “coherent,” “fast,” “stress,” “mental health,” “HRVB,” “mindfulness,” “anxiety,” “depression,” and “mental disorders”.

## 3. The Influence of Chronic Stress on Mental Health

One in eight people worldwide suffers from some mental disorder (MD), which ranks among the leading causes of disease and disability globally [25]. Modern lifestyle plays a central role in the development of MD [26,27]. Key factors affecting mental health include uncertainty and the anticipation of future stress, intensified by constant access to distressing global news [28,29]. The COVID-19 pandemic has markedly increased stress levels, further straining the mental health of the entire population and underscoring the urgent need for robust and accessible psychological interventions [30].

**Figure 1:**
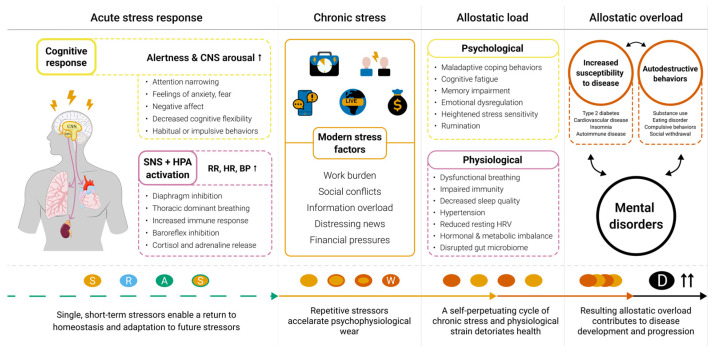
The development of allostatic overload. Note: this figure illustrates how acute stress responses progress into chronic stress, leading to allostatic load and, if unresolved, culminating in allostatic overload. The first panel highlights the effects of acute stress on both physiological responses and cognitive changes. The second panel presents modern stress factors that contribute to cumulative wear and tear on health. The third panel depicts the resulting allostatic load, while the final panel illustrates the ultimate consequence of allostatic overload—an increased risk of mental disorders and disease progression. Abbreviations: BP (blood pressure), CAN (central autonomic network), CNS (central nervous system), HPA (hypothalamic-pituitary-adrenal axis), HR (heart rate), HRV (heart rate variability), RR (respiratory rate), SNS (sympathetic nervous system), S (stress, yellow circle), R (rest, blue circle), A (adaptation, green circle), W (wear and tear, orange circle), D (disease progression, black circle), and ↑ (increase). Selected artwork used in this figure (lung, heart, brain, body outline, kidney) was adapted from pictures provided by Servier Medical Art (Servier; https://smart.servier.com/ accessed on 17 March 2025). Licensed under a Creative Commons Attribution 4.0 Unported License.

Contemporary social environments expose individuals to a much broader range of stressors compared to ancestral times, overwhelming human cognitive and coping capacities, leading to heightened psychological strain (Figure 1). Unlike past threats, which were rather tied to immediate physical danger, modern stressors primarily arise from professional, financial, social, and lifestyle pressures [31,32,33,34]. Consequently, many psychological disorders might be seen as diseases resulting from evolutionary mismatch [26,35,36]. Anxiety, depression, and various other mental conditions may once have served as adaptive responses to specific challenges or threats, but in today’s stress-laden world, they often become intensified and maladaptive [37,38,39,40].

Basically, stress is a normal psychophysiological response to conditions that disrupt the body’s balance and homeostasis [41,42]. Acute stress activates the “fight-or-flight” response, a fundamental survival mechanism. Through increased activation of the sympathetic nervous system (SNS) and the hypothalamic-pituitary-adrenal (HPA) axis, acute stress response triggers a cascade of physiological reactions that optimize physical and mental performance [43,44,45,46]. However, when stress-related changes persist over time, chronic stress emerges as a pathological state where negative emotional and physiological reactions continue even after the initial stressor is no longer present [43,47]. Prolonged stress disrupts the body’s balance, leading to the dysregulation of vital physiological processes, including sleep and metabolism, as well as cardiovascular, immune, and endocrine functions [43,48,49,50]. The impact on the brain and cognitive function is also profound and multifaceted (Figure 1). Chronic stress has been linked to structural brain damage—affecting memory, emotional regulation, and decision-making [51,52,53]. Consequently, chronic stress is a crucial factor in the development and progression of both physical and mental lifestyle diseases [51,52,54,55].

The cumulative burden of chronic stress and life events that exceeds an individual’s coping capacity, leading to unhealthy behaviors and increased susceptibility to disease, is known as allostatic overload (Figure 1). Manifesting in irritability, feeling overwhelmed, and difficulty relaxing, allostatic overload results from prolonged activation of the stress response, which suppresses the activity of the parasympathetic nervous system (PNS) and disrupts homeostasis [56,57,58]. Chronic stress may also induce dysfunctional breathing (DB), a condition characterized by persistent and detrimental alterations in natural biomechanical breathing patterns, most commonly associated with hyperventilation, thoracic breathing, and diaphragm inhibition. The resulting symptoms of respiratory effort and dyspnea co-occur in numerous pulmonary and cardiac conditions and have been associated with reduced quality of life [59,60].

Chronic stress affects a vast number of people across all age groups, populations, and professions, imposing a substantial economic burden [61,62,63,64]. Moreover, stress is increasingly recognized as an issue impacting both patients and healthcare professionals, making it an emerging public health concern [65,66,67]. Therefore, there is an urgent need to identify stress management methods that are safe, affordable, easy to use independently, and, most important, scientifically proven to be effective. Such interventions could offer valuable alternatives to pharmacotherapy and other methods that carry the risk of side effects [23,68].

## 4. Breathwork Mechanism of Action in Theory and Research

### 4.1. Respiratory Sinus Arrhythmia

A key biomarker of chronic stress and allostatic load is heart rate variability (HRV) [69,70]. HRV is a measure of the variation in the time interval between consecutive heartbeats, representing a natural physiological phenomenon influenced by the overlapping activity of both branches of the ANS [71,72]. HRV is an indicator of health and resilience, as it reflects the body’s complex physiological responses to a wide range of stimuli [73,74]. Chronically reduced HRV indicates a diminished ability to adapt to internal and environmental factors, serving as a marker of ANS dysfunction. Low HRV values are predictors of many lifestyle diseases, poor mental health, and all-cause mortality [69,73,75,76].

One fundamental mechanism contributing to HRV is Respiratory Sinus Arrhythmia (RSA). RSA refers to the natural fluctuations in heart rate (HR) during the breathing cycle, reflecting how respiration influences vagal outflow through both central and peripheral regulatory mechanisms [77,78,79]. HR increases during inhalation as a result of parasympathetic inhibition. Conversely, exhalation restores vagal activity, causing HR to decrease [78,79].

**Figure 2:**
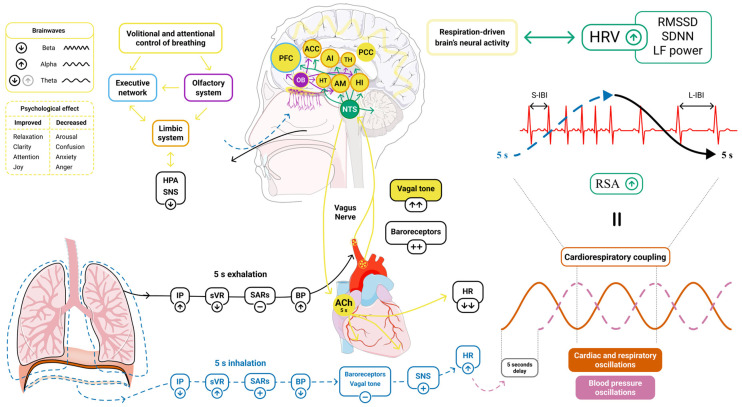
A detailed representation of the theoretical physiological and neurophysiological pathways involved in coherent breathing. Note: Current proposed mechanisms of influence on the brain and heart through resonance/coherent breathing are based on the formation of cardiorespiratory coupling and interaction with key neuronal structures involved in emotional regulation. Rhythmic breathing at a frequency of 0.1 Hz is the basis for the formation of coherence between heart, breathing, and blood pressure oscillations. Inhalation (indicated by blue arrows), based on mechanical and neural reactions, is associated with an acceleration of the heart rate. Prolonged exhalation (black arrows) causes strong engagement of baroreceptors and increased activity of the vagus nerve, which has the potential to maximally affect the heart’s pacemaker due to prolonged hydrolysis of acetylcholine in vagal efferents. Volitional coherent breathing may also impact brain structures via the nucleus tractus solitarius and olfactory bulb stimulation, affecting limbic and paralimbic regions and the central autonomic network, which leads to the propagation of oscillatory activity in the cerebral cortex. EEG recordings often show an increase in alpha-wave power, accompanied by a decrease in beta- and theta-wave power (though in some cases, theta activity may also increase). There is also a functional connection between the NTS and the locus coeruleus, which is not shown in the figure for the sake of clarity. The overall psychophysiological effect includes higher heart rate variability, a subjective sense of relaxation, and reduced arousal. Selected artwork used in this figure (lungs, heart, brain) was adapted from pictures provided by Servier Medical Art (Servier; https://smart.servier.com/), accessed on 17 March 2025. Licensed under a Creative Commons Attribution 4.0 Unported License. Abbreviations: Upward arrows (↑) indicate an increase, downward arrows (↓) indicate a decrease, a minus sign (–) denotes reduced activity, and a plus sign (+) denotes increased activity. Abbreviations: ACC (anterior cingulate cortex), ACh (acetylcholine), AM (amygdala), AI (anterior insula), BP (blood pressure), HI (hippocampus), HPA (hypothalamic-pituitary-adrenal axis), HR (heart rate), HRV (heart rate variability), HT (hypothalamus), IP (intrathoracic pressure), L-IBI (long interbeat interval), LF power (low-frequency power), NTS (nucleus tractus solitarius), OB (olfactory bulb), PCC (posterior cingulate cortex), PFC (prefrontal cortex), RMSSD (root mean square of successive differences), RSA (respiratory sinus arrhythmia), SARs (slowly adapting pulmonary receptors), S-IBI (short interbeat interval), SDNN (standard deviation of NN intervals), SNS (sympathetic nervous system), sVR (systemic venous return), TH (thalamus).

RSA is influenced by central respiratory rhythms in the brainstem and feedback mechanisms, including pulmonary stretch and baroreflex inputs, which work collectively to adapt RSA to changing respiratory and cardiovascular conditions [77,80,81]. Slow-paced breathwork techniques have been shown to amplify these processes, greatly improving RSA and enhancing parasympathetic function (Figure 2).

One of the recently proposed models suggests that the activation of slowly adapting pulmonary receptors (SARs) during deep inhalations contributes to vagal tone inhibition and increases sympathetic activation. Conversely, the cessation of SARs activity combined with baroreceptor stimulation during slow exhalation has been associated with a rebound in parasympathetic activity, which may promote positive HRV changes and a relaxation response [13,82]. According to the proposed model, the rhythmic engagement of SARs and baroreceptors during slow, deep breathing has been hypothesized to support the synchronization of respiratory and cardiovascular rhythms, optimizing ventilation-perfusion matching and reducing stress-related impact [82,83]. SARs and baroreceptors may also be integral to the beneficial psychological effects of breathwork, as their afferent signals to the nucleus of the solitary tract (NTS) have been proposed to modulate the central autonomic network, which is interconnected with higher cortical areas (Figure 2) [13,82,84].

### 4.2. Heart Rate Variability

A substantial portion of the positive effects of breathwork on stress and health has been attributed to improvements in HRV parameters [12,13,85,86]. HRV can be assessed through time and frequency domain analysis [71]. In the case of time-domain parameters, the Root Mean Square of the Successive Differences (RMSSDs) is considered a valid marker of parasympathetic activity that is not affected by respiratory influences [12,87,88]. However, some evidence shows that RMSSD may not reliably capture key features of HRV during slow deep breathing, indicating that measuring RSA might offer a more nuanced insight into parasympathetic reactivity [79,89]. The standard deviation of normal-to-normal interbeat intervals (SDNN) is also a commonly used parameter in breathwork studies during short-term HRV recordings [87].

Regarding the interpretation of frequency domain analysis, it remains somewhat unclear and often yields contradictory results [13,90]. The relationship between various HRV frequency bands, the activity of different branches of the ANS, and the influence of other physiological factors is still not fully understood [71]. Particularly controversial is the interpretation of the low-frequency (LF) to high-frequency (HF) HRV ratio as an indicator of sympathovagal balance, which is currently discouraged in studies involving slow-paced breathwork measurements [71,89,91,92]. With a breathing frequency below 9 bpm (breaths per minute), LF alone appears to be a reliable marker of vagal activity [12], further confirmed by a study comparing pharmacological sympathetic and parasympathetic blockades [86]. HF represents parasympathetic activity at spontaneous breathing rates, which may explain the ambiguity behind the LF/HF ratio [89].

Evidence shows that vagally mediated HRV parameters, including RMSSD, SDNN, and LF, improve during slow deep breathing, suggesting that vagus nerve involvement may be the unifying pathway behind the physiological benefits of various breathwork techniques [12,93,94,95,96,97]. Approximately 80% of vagal fibers are afferent, and when stimulated by breathing, they have been thought to alter the activity of numerous brain areas via connections with the NTS. The NTS, which receives signals almost exclusively from the vagus nerve, relays them to various functionally connected brain regions central to cognitive functioning, mood regulation, emotional processing, and autonomic control [98,99,100,101] (Figure 2). These regions include the amygdala, anterior cingulate cortex, thalamus, hypothalamus, insular cortex, hippocampus, orbitofrontal cortex, the largest serotonergic nucleus (dorsal raphe nucleus), and the primary noradrenergic nucleus (locus coeruleus) [82,99,100,101,102,103,104].

For techniques based on high-frequency breathing (fast breathing), such as Kapalbhati pranayama [105], an inverse reaction of HRV parameters and vagal withdrawal has been observed. Characterized by a reduction in SDNN, RMSSD, and HF power, along with an increase in LF power, fast breathing seems to primarily involve sympathetic activation [105,106] but may ultimately lead to a subsequent parasympathetic rebound after the intervention [106].

### 4.3. Cardiorespiratory Coupling

Coherent breathing, performed at a characteristic rate of ~6 bpm, is currently considered the most effective breathing technique for enhancing HRV. At this frequency (0.1 Hz), temporal coherence has been observed among three main oscillators: variations in HR, respiration, and blood pressure (BP) [103]. It is hypothesized that a key phenomenon here is baroreflex resonance, a negative feedback mechanism in which HR and BP fluctuate in antiphase within a closed-loop system, creating cardiac oscillations at a frequency of approximately 0.1 Hz [103,107,108]. These oscillations, however, diminish over time during spontaneous breathing due to a natural delay in BP response to HR change caused by vascular dynamics (a detailed description of this phenomenon can be found in [103]). When respiratory rate adopts ~6 bpm, the delay diminishes, and coupling between cardiac and breathing rhythms has been demonstrated to occur, sustaining baroreflex resonance and reinforcing cardiac oscillations [103,107]. Additionally, slow breathing provides sufficient time (~5 s of exhalation) for acetylcholine produced by vagal efferents to be fully released and hydrolyzed, potentially imposing a maximal vagal effect on the heart. As a combined result, RSA amplitude and HRV are maximally elevated (Figure 2) [103]. Interestingly, Hinterberger et al. have also demonstrated the highest synchronization of slow cortical potentials in the frontal and central brain regions at a breathing rate of 6 bpm compared to other rates. Thus, coherent breathing may synchronize not only respiration, HR, and BP, but also brain activity [109].

Closely related to coherent breathing is resonant breathing, although these terms are often used interchangeably. A respiratory rate of 0.1 Hz could be generally referred to as the resonant frequency [103]. Nevertheless, each individual has slightly different delays in the reaction of BP and HR changes, which enables the determination of a personal baroreflex resonance frequency. By using HRV biofeedback (HRVB), it is possible to identify the frequency at which PNS activity parameters are at their highest [107]. HRVB appears to be a more personalized intervention, as it focuses on finding the individual’s specific breathing frequency that creates an ideal synchrony between the cardiovascular and respiratory systems [97,103,110].

### 4.4. Breath-Brain Communication

Breathing is essential for brain function. By providing rhythmic sensory input, breathing synchronizes cortical activity across multiple brain regions, extending beyond those directly linked to respiration [111,112]. More than 30 years ago, early electroencephalographic (EEG) research observed that various pranayama techniques, as well as simply slowing the respiratory rhythm, change the dynamics of CNS activity [113,114]. Since then, multiple EEG studies have associated breathwork practices, such as HRVB, diaphragmatic breathing, and paced breathing, with calmness and reductions in arousal, as indicated by decreased beta power [115,116,117,118], alpha variability [113], and frontal alpha asymmetry [119]. Decreases in theta power [13] and substantial increases in alpha power [13,104,115,118,120] in brain regions associated with ANS modulation, stress regulation, and relaxation are also frequently reported [115,118,119]. Some findings also show increased frontal theta power during slow breathing [117,118,119]. Increased theta activity has been associated with focused attention and deeper meditative states, which enhance parasympathetic function, promote relaxed alertness, and help alleviate symptoms of anxiety [117,118,119]. It is noteworthy that the phrenic nerve, which innervates the diaphragm, has sensory fibers that project to key brain areas, including the limbic system. External phrenic nerve stimulation in critically ill patients enhances brain connectivity and cortical activity in the frontal, temporal, and parietal cortices, mirroring the effects of diaphragmatic breathing and suggesting an additional pathway through which breathwork may influence cognition [121].

Unfortunately, there is a lack of research on the impact of higher breathing frequencies on brain function. A recent study [122], which employed a high-density EEG during fast breathing (1 Hz), observed increased alpha power and a notable rise in gamma oscillations across the fronto-temporal-occipital brain regions. The authors suggest that these changes may be associated with enhanced cognitive abilities, improved attention and emotional regulation, and also promote higher cognitive states [122]. Herrero et al., in their intracranial EEG study, demonstrated that cortical and limbic neuronal activity in the gamma band is phase-locked to specific phases of the breathing cycle [112]. It was observed that when participants volitionally increased their breathing rate, respiration-locked oscillations also increased in frequency and spatial extent. In contrast, during spontaneous automatic breathing, both phase-locking and coherence were reduced.

Notably, the olfactory bulb emerges as an important structure involved in propagating respiration-locked oscillations [112]. This observation is particularly intriguing, as a frequently discussed factor in the context of breathwork’s impact on brain activity and the ANS is the preferred breathing route. Nasal breathing is traditionally considered superior to mouth breathing in many breathwork techniques and meditative practices [13,123,124]. Mechanical stimulation of the olfactory epithelium affects key brain regions, including the prefrontal cortex, hippocampus, and amygdala [123,125,126]. The effects of nasal breathing are independent of mouth and thoracic respiration [13,126]. Nasal breathing facilitates an increase in theta, delta, and high-beta power and synchrony, suggesting heightened activation of the olfactory system as a potential explanation for the altered states of consciousness often experienced during meditation and breathwork [123,125,126]. Nasal breathing promotes synchronization of brain circuits in the piriform cortex, medial prefrontal cortex, posterior areas, and limbic-related brain regions, with this effect diminishing significantly when shifted to mouth breathing [123,126]. Rhythmic activation of olfactory pathways has been associated with memory-related hippocampal rhythms and synchronization of brain activity, fostering calm and emotional balance [112,127,128,129].

Another highly influential factor in the functioning of the CNS may be the sole act of observing or counting breathing [130]. Mindful breathing, where one attentively focuses on breathing sensations, is central to meditative practices and many breathwork techniques [131,132]. However, breath awareness and voluntary breath control activate different but overlapping neural circuits. Attention to breathing enhances respiration-locked oscillations, particularly in the anterior cingulate cortex, while volitional breathing engages broader cortical and limbic regions [112].

In summary, breathwork not only has been shown to modulate ANS activity through vagal stimulation but also has been associated with enhanced neural oscillation coherence, potentially improving communication between brain regions involved in emotional regulation, behavior, and cognition [123,126]. The dynamic interplay between the executive function network and both vagus nerve activity and the limbic system appears to be particularly important. The prefrontal cortex, which is integral to stress regulation and directly linked to HRV, plays a central role in mediating the effects of breathwork (Figure 2) [15].

## 5. The (Paradoxical) Diversity of Breathing Techniques

There are countless ways to alter one’s respiration, making breathwork encompass a wide array of breathing techniques and exercises, each emphasizing different theoretical mechanisms of action. Nearly every breathing technique is attributed to its distinct properties and effects, which contribute to the high heterogeneity of research findings (Table 1). This variability complicates the task of conducting comparative analyses of these techniques. Additionally, breathwork studies often face numerous challenges, such as methodological inconsistencies, small sample sizes, and a lack of standardized protocols.

Consequently, most recent research has focused on identifying the actual mechanisms through which breathwork may produce its positive effects. In the following subsections, we will critically explore key variables that are considered to shape and differentiate specific breathing techniques. We propose, in the context of long-term mental health outcomes, that various breathwork techniques are closely linked through their shared influence on ANS modulation and cognitive factors, ultimately converging toward similar psychological benefits. However, this does not imply that there are no differences between techniques or their physiological mechanisms of action, especially in the short term. Rather, emerging evidence suggests that the final, long-term subjective experiences and psychological impact most commonly associated with relaxation and stress reduction may be similar for most techniques [16,133,134].

**Table 1:** A selection of the most recognized breathing techniques demonstrating the diversity of breathwork techniques, research approaches, and potential mechanisms of action. Some techniques are not included due to limited research.

Examples of Techniques with short description	Research, reference	Benefits of practice 1. Psychological 2. Physiological	Proposed mechanisms and pathways of action	Limitations
Breathing Pace				
Fast breathing				
**Kapalbhati pranayama;** **Skull** **shining breath** **;** **Breath of** **fire**	Within-subject design, immediate effects in yoga practitioners [105]; RCTs [106,122]	Feeling of joy, energizing [105,106]: Initial SNS activation (↑ HR, ↑ BP, ↑ LF) with PNS withdrawal (↓ HF, ↓ RMSSD, ↓ SDNN); subsequent PNS rebound (↑ HF, ↑ RMSSD, ↓ HR) [105,106]; ↑ alpha waves power in frontal and temporal brain regions, ↑ gamma wave power in frontal, temporal, and occipital regions [122]	Beneficial modulation of ANS: - SNS activation due to rapid breathing - post-intervention PNS dominance (strong vagal reactivation) Strong exhalations lead to ↓ CO_2_ and a loss of hypercapnic drive ↑ alpha activity suppresses irrelevant sensory inputs through top-down regulation of attention Strong olfactory stimulation impacts limbic and cortical areas	Small sample sizes, very short interventions (5 min) [105,106], small sample sizes restricted to healthy young males, and lack of post-intervention measurements [122]
60 to 120 bpm; short and forceful exhalations with a strong engagement of abdominal muscles and passive inhalations				
**Conscious connected breathing**	Within-subject design in healthy adults during practice [135]	↓ negative affect states (tension, confusion, depression, anger), ↑ self-esteem ↓ delta, ↓ theta power in frontotemporal and parietal regions, ↓ low-beta power in parietotemporal areas, ↑ gamma power	Changes in brain activity related to subjective feelings of altered states of consciousness (resembling moderate-to-high dose psilocybin experiences)	Small sample size of experienced practitioners, lack of control group
Deliberate, fast, deep cyclical breathing without a pause between inhale and exhale, high diaphragm engagement				
Slow Breathing				
**Coherent breathing**	Within-subject design comparing acute effects of two breathing ratios [97]; fMRI study with experimental manipulation of breathing rate and oxygen levels [136]; experiment during acute stress and paced breathing [137]	↑ feelings of relaxation [97]; ↑ alertness [136]; ↑ SDNN, ↑ LF [97]; ↑ tidal volume, increased activity of multiple brain and brainstem areas n (hypothalamus, hippocampus, insula, dorsal pons) [136]; increased physiological coherence measured in signals synchronization (derived from HRV, PTT, and respiration) [137]	Cardiorespiratory coupling ↑ cardiac vagal activity (high baroreflex and vagal afferent activity) Recruitment of brainstem and cortical structures involved in physiological response Induction of a contemplative mental state	Small sample sizes, lack of actual RR and breathing ratio control [97]; discomfort associated with experimental settings, confounding non-neural effects [136]; important markers of stress (cortisol) were not measured [137]
~5.5 bpm with equal inhalation and exhalation durations				
**Resonant breathing;** **HRV biofeedback**	RCT involving young adults practicing for 4 weeks [138]	↑ cognitive performance, ↓ perceived stress Improvements in resting HRV parameters including SDNN, pNN50, LF, LF/HF and total power	High baroreflex resonance and increased vagal tone improve sympathovagal balance Improved functional relationship between the prefrontal cortex and the central autonomic network	Limited sample of young male adults
Breathing at individualized resonance frequency focused on maximizing RSA (~ 6 bpm)				
**Diaphragmatic breathing**	Systematic review of one RCT and two quasi-experimental studies [68]	↓ self-perceived stress, ↑ sustained attention, ↓ negative affect ↓ RR, ↓ salivary cortisol levels, ↓ BP	↑ vagal activity, ↓ SNS activity, HPA axis modulation, improved attention and cognitive functions	Outcome measures and methodological heterogeneity, low quality of evidence, small sample sizes
Involved deep inhalation concentrating on maximal diaphragm engagement and lateral rib movement (3–7 bpm)				
**Zen *tanden ***breathing****	Within-subject quasi-experimental design, participants unexperienced of Zen meditation [104]	Feeling of vigor-activity, ↓ anxiety ↑ urinary serotonin levels, ↑ high-frequency alpha activity	Activation of serotonergic neurons in the dorsal raphe nucleus contributes to the observed EEG changes	Small sample size, narrow range of measured parameters
Prolonged rhythmic contraction of abdominal muscles during exhalation (3–4 bpm)				
**Okinaga, extreme prolongation of expiration**	Within-subject quasi-experimental design, 5 male experienced practitioners, acute effects of 31-min intervention [118]	↑ relaxation ↑ theta and alpha waves, ↓ beta waves, ↓ HF (although there is inconsistency between the text and figure representing HF values over time), ↓ in LF/HF ratio	PNS dominance during practice and decrease in beta power ↑ frontal theta power potentially indicating greater focused attention ↑ alpha 2 power linked with relaxation	Small sample size of 5 experienced practitioners; EEG limited to forehead; ambiguity in LF/HF ratio interpretation
Emphasis placed on long exhalations (~1 bpm)				
Breathing Pattern				
**Box breathing;** **Tactical breathing;** **Sama Vritti pranayama**	Remote, 4-week long RCT comparing breathwork techniques to mindfulness [139]; Within-subject design comparing box breathing to prolonged expiration (2 s I, 8 s E) during stress task [140]	Improvements in physiological arousal and mood, ↓ state anxiety and negative emotions [139]; ↓ RR [139]; ↓ physiological arousal (↓ HR, ↓ RR) compared to the second prolonged exhalation both during training and stress coping task [140]	Modulation of brain function by vagus nerve pathways, enhancement of interoceptive awareness, enhanced self-regulation and stress perception through modulation of brain regions involved in emotional processing The diving response increases PNS activity during breath holds Focused attention associated with better distraction from stressors	Remote study with daily self-reports [139]; lack of a control group without breathing, low sample size, short training duration [140]
Equal durations of inhalation, breath hold, exhalation, and breath hold, e.g., 4-4-4-4 s; Typically used in high-stress professions (e.g., military)				
**4-7-8 breathing**	Quasi-experimental study, immediate effects after one intervention [141]; randomized controlled experimental design with a pretest–posttest control—patients after bariatric surgery [142];	↓ state anxiety levels [142]; ↑ PNS activity: ↓ SBP, ↓ HR, ↑ HF, ↓ LF/HF; ↓ sympathetic activity: ↓ LF, ↓ SDNN [141]	Enhanced parasympathetic activity post-intervention due to: Low inhale/exhale ratio and slow breathing pace (3 bpm) resulting in ↑ RSA amplitudes ↓ peripheral chemoreceptor stimulation due to increased arterial oxygen saturation during breath holds Attention modulation and relaxation through oxygenation and CO_2_ expulsion	Small sample size with high female-to-male ratio, ambiguity in HRV interpretation [141]; small sample size, narrow clinical population [142];
4-7-8-0 pattern: 4 s inhalation, 7 s breath hold, 8 s exhalation				
**Cyclic** **sighing**	Remote, 4-week long RCT comparing breathwork techniques to mindfulness [139]	↑ positive affect, ↓ negative emotions ↓ RR	ANS regulation by the enhancement of vagal tone via extended exhalations	Remote nature of study, daily self-reports
(1 + 0.25):2 ratio pattern—double inhale with long exhalation				
Breathing Route				
**Slow nasal/mouth** **breathing**	Randomized, within-subjects crossover design; study comparing mouth vs. nasal breathing in 12 experienced meditators [123]	Improvements in nasal compared to mouth breathing: Perception of being in an altered state of consciousness, ↓ anxiety levels, ↑ positive affect (heightened feeling of joy); Comparable effects on HRV and brain rhythms modulation in posterior areas (indicating similar vagal contribution); ↑ theta, ↑ delta, ↑ high-beta powers and connectivity in prefrontal and frontal areas unique to nasal breathing	Nasal breathing stimulates mechanoreceptors within the olfactory epithelium—the olfactory bulb projects to the piriform cortex and (via the mediodorsal thalamic nuclei) to the prefrontal cortex, amygdala, and other cortical and limbic areas relevant for emotional regulation Heightened theta and high-beta coupling in default mode network areas supports both local and long-range communication between brain regions Slowing of cortical rhythms (delta-theta) important for non-ordinary states of consciousness	Low sample size, healthy meditative practitioners (limiting applicability to clinical and general populations), unequal gender distribution,
2.5 bpm nasal breathing with 6-6-6-6 s pattern				
**Pursed lip breathing**	RCT, hypertensive urgency patients [143], systematic review and meta-analysis of 15 RCTs combining pursed lip and diaphragmatic breathing in COPD patients [144]; quasi-experimental study, COPD subjects [145]	2. ↓ SBP, ↓ DBP, ↓ HR [143]; improved pulmonary function (FEV1, FVC, FEV1/FVC ratio) and exercise capacity (6-min walk test distance) [144]; ↑ pulse oxygen saturation, ↑ RMSSD, ↓ RR [145]	↑ PNS activity influences autonomic cardiac modulation Time counting increases mindfulness buffering anxiety and stress ↑ expiratory resistance leads to ↓ airway resistance and residual volume, enhancing lung emptying, gas exchange, ventilation efficiency, tidal volume, and expiratory muscle recruitment	Limited generalizability of the findings, low methodological quality, narrow clinical populations [143,144], small sample size, lack of control group [145]
Inhalation through the nose (2 s), followed by slow, controlled exhalation through puckered or pursed lips (4 s), involving number counting. Typically used in older patients with COPD				
**Sheetali pranayama;** **Cooling breath**	Parallel-group randomized trial comparing 12 weeks of Bhramari vs. Sheetali, hypertensive patients [146]; single blinded RCT, hypertensive patients, 3 months practice [147]	2. ↓ serum cortisol, ↑ serum nitric oxide, ↓ BP, improvements in physical fitness (sit-to-stand test) and quality of life [146]; improvements in various BP variables, ↑ HRV parameters (↑ SDNN, ↑ RMSSD, ↑ pNN50, ↑ HF, ↓ LF, ↓ LF/HF ratio) [147]	↑ PNS activity through vagus nerve and baroreceptors stimulation, high influence on HPA axis, improved baroreceptor sensitivity	Limited sample size, lack of control group, hypertensive elderly population limiting generalizability [146]; possible placebo effect [147]
Involves curling the tongue to form a “straw,” inhaling fully through the rolled tongue, closing the mouth, holding the breath briefly, then exhaling slowly through the nose				
**Alternate nostril breathing;** **Anulom-Vilom pranayama; Nadi-shodhana pranayama**	Systematic review including 44 RCTs [148]	Enhanced cognitive performance, improved memory; ↓ self-reported anxiety and depression scores after regular practice Improved autonomic modulation and increased vagal activity: ↑ HRV parameters, ↓ SBP, ↓ DBP, ↓ HR, ↓ pulse rate; improved pulmonary function parameters (peak expiratory flow rate, forced expiratory flow, FVC, FEV1, maximum voluntary ventilation, vital capacity)	Alternating nostril flow restores ANS balance—right nostril associated with ↑ SNS activity, left nostril with ↑ PNS Mechanical receptors in nasal mucosa stimulate hypothalamic pathways influencing autonomic balance ↑ vagus nerve activity promotes neuroplasticity through release of acetylcholine, epinephrine, and brain-derived neurotrophic factor Breath awareness promotes mindfulness effects: improving self-regulation, stress coping strategies, cortical-subcortical connectivity, ↓ stress-related physiological responses	Methodological heterogeneity including wide variation regarding nostril sequence, breath retention duration, and I:E ratio, limited studies on clinical populations, diverse outcome measures, lack of standardization
Involves breathing through one nostril at a time while manually closing the other nostril. The technique has variations in nostril switching, inhalation/exhalation duration, and breath retention				
Breathing Sound				
**Ujjayi pranayama;** **Ocean breath**	Within-subject design comparing various breathing paces with or without ujjayi in young healthy participants [149]; within-subject design of immediate effects among university students [150]	↓ anxiety scores, ↑ sustained and selective attention [150]; ↑ oxygen saturation, ↑ HR, ↓ chemoreflex sensitivity, ambiguous effect on baroreflex sensitivity [149]	Contraction of the glottis muscles increases the intrathoracic pressure intensifying vagal activity, baroreflex sensitivity, and oxygen saturation Increased respiratory effort potentially ↑ SNS activation, counterbalancing some effects of PNS activation Improved attention due to enhanced cognitive control and response inhibition to unnecessary stimuli, regulation of brain regions involved in emotion processing	Small sample size, narrow range of parameters, lack of HRV measurements [149]; small sample size with experienced practitioners (2 years of pranayama practice) [150]
Deep breathing involving slight contraction of the glottis to produce a gentle hissing (ocean) sound during nasal inhalation and exhalation; typically slow-paced with equal I:E ratio				
**Bumble bee breath** **;** **Bhramari pranayama**	Pilot, cross-sectional, observational study with Holter-based HRV measurement comparing various activities [151]; systematic review [152]	↓ stress index [151]; feeling of “bliss” [152] ↑ in total HRV power and SDNN compared to other activities (physical activity, emotional stress, sleep); ↓ HR during breathwork compared to stress and physical activity; high LF/HF ratio [151]; ↓ BP, ↑ gamma wave activity [152]	Prolonged exhalations enhance RSA, ↑ PNS and ↓ SNS activation Generation of low-frequency respiratory oscillations (acoustic vibrations), which may stimulate baroreflex sensitivity and optimize heart-lung coupling ↑ nasal nitric oxide levels during the humming Potential of sound vibrations in influencing cerebrospinal fluid flow	Small sample size, lack of placebo control group [151]; lack of RCTs, high heterogeneity, no cross-cultural validation, general low quality of analyzed studies [152]
Generation of humming sound during nasal exhalation, ~4–6 bpm, with exhalation twice longer than inhalation				
Combined Techniques				
**Bhastrika pranayama** **;** **Bellows breath**	RCT, fMRI analysis of brain activity and psychological assessment after regular 30 days pranayama practice [153]	↓ state anxiety, ↓ negative affect, along with ↑ positive affect and awareness; enhanced emotional regulation, particularly during exposure to emotionally laden stimuli; Modulation in activity and connectivity of neural circuits (amygdala, anterior cingulate cortex, anterior insula, and prefrontal cortex)	↓ functional connectivity between the right anterior insula and lateral prefrontal cortex correlated with reduced anxiety ↑ activity in the right amygdala and anterior insula was linked to the least reduction in negative affect and anxiety Activity changes in anterior cingulate cortex and anterior insula related to emotional awareness	Small sample size, lack of ANS assessments, daily practice not monitored, additional maneuvers along breathing
30 cycles: combination of 30 rapid breaths, followed by Surya Bedhana (inspiration through right nostril—breath hold—expiration through left nostril) with muscle activation				
**Sudarshan kriya yoga** **(SKY) breathing**	Pilot feasibility study with a single arm pre-post design, 4-days practice and 40 days follow-up [154]; RCT, 6-week intervention, military veterans with PTSD [155]	↓ stress levels, ↓ anxiety, ↓ depression, and improvement in resilience, life satisfaction, and sleep quality score [154]; improved self-reported emotion regulation [155] ↑ HR min-max, ↑ normalized HF, ↓ LF/HF; nonsignificant improvements of other HRV parameters (RMSSD, SDNN, absolute HF-HRV, LF) [155]	Improved ANS regulation and balance Enhanced emotional regulation Changes in RSA and RR Parasympathetic activation	Lack of a control group and randomization, selection bias, low follow-up sample [154]; poor data quality, ambulatory HRV measurement [155]
~30 min. long typically consisting of 4–5 breathing techniques varying in pace (e.g., ujjayi, ANB, Bhastrika, cyclical SKY breathing with ascending pace)				
**Wim Hof breathing method**	RCTs [156,157]; Systematic review [158]	Decreases in perceived stress [157]; increased energy, mental focus, and attention [156], psychically challenging [158] Anti-inflammatory effect (↑ interleukin-10, ↓ pro-inflammatory cytokines), ↑ epinephrine levels [158] Combined Wim Hof method (with cold water exposure and meditation) lead to superior results than breathing alone	↑ pH levels, ↑ oxygen saturation, ↑ HR, increase in plasma epinephrine levels Strong SNS activation Epinephrine release, and diminished innate immune response due to decrease in pro-inflammatory cytokines	Low sample size, large proportion of males, potential influence of placebo effect due to expectations and surroundings, high risk of bias across studies [158]
Cyclic hyperventilation, 30–40 fast and deep forceful nasal inhalations with light exhalations through mouth, followed by long breath hold (60–120 s) with subsequent inhalation and shorter (15 s) breath hold				

Abbreviations: ANB (alternate nostril breathing), ANS (autonomic nervous system), BP (blood pressure), bpm (breaths per minute), CO_2_ (carbon dioxide) COPD (chronic obstructive pulmonary disease), DBP (diastolic blood pressure), E (exhalation), EEG (electroencephalography), FEV1 (forced expiratory volume in one second), FVC (forced vital capacity), fMRI (functional magnetic resonance imaging), HF (high frequency HRV), HPA (hypothalamic-pituitary-adrenal axis), HR (heart rate), HRV (heart rate variability), I (inhalation), LF (low frequency HRV), pNN50 (percentage of successive RR intervals that differ by more than 50 ms), PNS (parasympathetic nervous system), PTT (pulse transit time), PTSD (posttraumatic stress disorder), RCT (randomized controlled trial), RMSSD (root mean square of successive difference), RR (respiratory rate), RSA (respiratory sinus arrhythmia), SBP (systolic blood pressure), SDNN (standard deviation of NN intervals), SNS (sympathetic nervous system), ↑ (increase), ↓ (decrease).

### 5.1. Breathing Pace

In breathwork research, special attention is given to slow-paced breathing, as its short-term physiological effects are considered a potential foundation for achieving long-term mental health benefits [13,21,95,159,160]. However, despite promising results, the high heterogeneity of study methodologies, the limited number of longitudinal studies with follow-ups, and the lack of comparisons to well-designed placebo controls all restrict the ability to draw definitive conclusions about the correlation between breathing pace, its psychophysiological mechanisms, and long-term mental well-being [13,90,103].

Recently, Fincham et al. conducted a rigorously designed, blinded, randomized controlled trial (RCT) comparing the effects of coherent breathing (5.5 bpm) on stress with a placebo intervention paced at spontaneous breathing frequency (12 bpm) [16]. The study recruited 400 adults from the general population who engaged in breathwork for 10 min daily over a four-week period. The authors did not inform participants of any potential advantages or superiority of a particular breathing technique, thereby eliminating the influence of specific expectations—an omnipresent factor in breathwork research [16]. Notably, after the intervention and at the four-week follow-up, participants in both groups demonstrated statistically significant improvements in self-reported measures of stress, anxiety, depression, and general well-being, with no significant differences observed between groups (only a significant effect of time was observed for both groups, *p* < 0.001). Despite the fact that coherent breathing appears to have the highest potential physiological impact (Figure 2), the largest and most well-designed study on this technique has not shown superiority over a well-designed placebo [16]. Similarly, Bentley et al., in their recent systematic review, found that in the context of slow-paced breathwork, specific breathing rates are not particularly crucial for long-term effectiveness in reducing stress and anxiety [20]. These findings highlight a critical gap in breathwork research, suggesting that the psychological effects of breathwork are likely influenced by numerous confounding variables.

The ambiguous long-term effects of breathwork extend even to research on two contrasting practices—fast and slow breathing. For instance, a 12-week study performed in India by Sharma et al. (*n* = 90), which compared two sets of three rapid and slow pranayama breathing techniques practiced for 30 min, three times a week, found similar reductions in perceived stress among healthcare students in comparison to inactive controls (*p* < 0.001), despite statistically significant within-group changes in certain cardiovascular parameters in favor of slow pranayama [134]. Furthermore, a recent, remote, rigorous, blinded RCT by Fincham et al., involving 182 participants, compared brief, remotely delivered fast breathwork with an active placebo control in the form of paced breathing at 15 bpm to assess the subjective mental health effects [133]. After three weeks of daily 20-min-long practice and a follow-up period, no significant differences were found between the groups in terms of stress levels, affect, anxiety, depression, mental well-being, or sleep-related impairment [133].

Unfortunately, there is currently little evidence explaining these findings. While fast and slow breathwork have been shown to exert their effects through distinct acute neurophysiological mechanisms, over time, they have also been observed to converge in their effects on ANS function and subjective mental well-being. The deliberate activation of the SNS in fast breathwork may contribute to subsequent positive ANS modulation and delayed positive stimulation of the PNS, fostering mental resilience to stress in the long-term [20,23]. One study on the immediate effects of fast-paced Kapalbhati pranayama, which included 50 healthy volunteers with a spontaneous breathing control group, has demonstrated that despite an initial surge in sympathetic activation and parasympathetic withdrawal during or immediately after practice, ANS activity shifts toward parasympathetic dominance 20 min post-exercise [106].

The comparison between different breathing paces, specifically fast and slow breathwork, remains a critical area of research that warrants further exploration, given the widespread popularity and long-standing traditions of these two contrasting breathing styles [161].

### 5.2. Breathing Ratio

One of the most debated aspects of breathwork, marked by numerous conflicting findings, is the impact of different breathing ratios (the proportion of inhalation (I) to exhalation (E)) in the case of slow breathwork [162]. Theoretical perspectives suggest that lower ratios, such as 1:2 (I to E), naturally seem to offer greater health benefits, particularly in the short-term [13,82,103,159,163].

Many studies have been conducted on this topic. The review by Meehan & Shaffer revealed somewhat contradictory results [162]. Three studies found no effects of I:E ratios, while others showed benefits: E = I (1 study), E > I (4 studies), or I > E (1 study). After identifying key methodological issues and challenges in these studies, the authors conducted their own RCT and compared the effects of 1:1 and 1:2 IE ratios (6 bpm) on various HRV measures [162]. In both the original and replication experiments, no differences were found in HRV time-domain and frequency-domain measures. The population of undergraduate students represents an important limitation of this study due to the absence of clinical populations or individuals exposed to high stress with low baseline HRV values [162].

Another single-blinded RCT with 79 participants compared two slow deep breathing techniques (4:2 and 4:4 IE ratios) to spontaneous breathing on stress markers in response to a virtual reality active shooter drill (VR-ASD)—an acute high-stress exposure [164]. The first group had an average of 5 bpm, while the second group had 10 bpm. Both slow breathing interventions, despite differing exhale durations, were equally effective in preventing significant increases in salivary α-amylase five- and thirty-minutes post-VR-ASD in comparison to the control group (*p* < 0.01). No differences were found between the two interventions regarding their effect on HR or state anxiety inventory scores [164].

Furthermore, Birdee et al. recently conducted a well-designed single-blinded RCT with 97 healthy individuals, comparing yoga-based slow breathing between the I = E group and the E > I group [159]. After 12 weeks (five breathing sessions per week with a 3 bpm pace), no statistically significant differences were found between the groups in terms of the positive impact on mental health and stress reduction. While improvements were observed in the psychological measure (PROMIS Anxiety questionnaire), no such changes were seen in the physiological one (HRV). Although not statistically significant, it should be noted that when comparing the groups, both measures showed better improvement in the E > I group [159]. To date, this is the only study of such high quality examining the effects of different exhale-to-inhale ratios on mental health and stress reduction over a longer period. However, further research is needed to validate these findings, especially considering methodological challenges such as sample size, control groups, and long-term follow-ups.

In summary, most recent robust studies with well-designed control conditions indicate that while different breathing techniques, varying in pace or breathing ratio/pattern, offer mental health benefits, the long-term effects are largely similar across these techniques.

### 5.3. Mindful Breathing

To what extent could the benefits of breathwork be attributed solely to breath awareness [16,103]? Both mindfulness-based techniques and those involving intentional breathing control appear fundamentally related.

To change the breathing rhythm, one must consciously control their breath, and conversely, mindful observation of the act of breathing decreases the frequency of respiration [89,112,165,166]. On the other hand, slow breathing techniques are associated with an increase in tidal volume to ensure adequate oxygen delivery [103,167]. To accommodate this demand, lower respiratory rates naturally involve greater diaphragm engagement, which provides extensive sensory feedback to the ANS and CNS regarding changes in breathing rhythm [21]. As a result, greater diaphragm engagement is observed during meditation, where a larger belly respiratory amplitude and a decreased respiratory rate are driven by increased activation of PNS pathways [166].

However, given its well-documented effects on key physiological processes described in the previous section, breathwork seems to offer greater potential to influence the body than mindfulness meditation [168]. For example, there is some ambiguity regarding the impact of mindfulness on HRV. Some studies have found positive effects [89,169,170], while others, including two meta-analyses comparing mindfulness to active controls, found no such effects [171,172]. In contrast, research strongly supports the positive effect of breathwork on modulating PNS activity [12]. Additionally, as breathwork represents a more active intervention, it may offer a greater sense of control and yield more immediate effects compared to a passive approach to mindfulness [139]. What about the long-term impact of these two practices on mental health?

Balban et al. recruited 114 participants to compare the effects of a daily 5-min mindfulness practice over 4 weeks with three different types of breathwork [139]. The results of this RCT indicated reductions in the state of anxiety and negative affect for both types of interventions. However, the breathwork groups showed significantly better improvements in positive affect and greater reductions in respiratory rate [139]. Surprisingly, the authors did not observe any statistically significant changes in HRV or resting HR in any group, despite daily monitoring. However, the remote nature of the study, the absence of further follow-ups, and the relatively small sample size are key limitations that restrict drawing definitive conclusions [139].

In another study, researchers compared HRVB and mindfulness interventions, both conducted daily for 6 weeks, to a wait-list control group. At the end of the intervention and at a 12-week follow-up, no group differences were observed across a wide range of psychological and physiological stress reduction measures. Although not statistically significant, the intervention groups appeared to be more effective overall, with more stressed individuals benefiting the most. It is important to note that this study had a small sample size and potential confounding factors, such as participants’ expectations and socio-psychological influences [173]. The findings of other studies regarding the differences in the long-term effects of these two practices also remain ambiguous [174,175]. Certainly, further research is needed to clarify how mindfulness and breathwork compare in their effects. While deliberate attention to breathing is a key factor for improving mental health through breathwork, its role as a central mechanism has yet to be confirmed.

### 5.4. The Route of Breathing

Another important aspect to consider is the preferred breathing pathway of a given technique. Some breathwork practices employ mouth breathing [147,176], although the majority utilize nasal breathing. In contrast, pronounced differences in brain activity have been observed between nasal and mouth breathing [112,123,126,177]. At rest, nasal breathing seems to offer modest cardiovascular benefits and shifts the autonomic balance toward parasympathetic dominance in the HRV frequency domain compared to mouth breathing [178]. It is important to emphasize that nasal breathing is the primary, natural respiratory pathway, and the negative effects of chronic mouth breathing, associated with the development of various disorders, are particularly evident in children [179,180].

Rhythmic, regular stimulation of the olfactory centers may be an important and, potentially, one of the commonly shared pathways through which various breathwork techniques exert their effects, alongside their influence on the vagus nerve [123]. Unfortunately, there is a lack of studies comparing these two breathing routes in the context of breathwork. In one study, Zaccaro et al. compared mouth versus nasal breathing (2.5 bpm) in 12 experienced meditators, aiming to highlight differences between the short-term effects of olfactory epithelium stimulation and those associated with vagus nerve activation [123]. As expected, both techniques had similar effects on ANS stimulation, as measured by HRV analysis. However, the groups differed regarding cortical activity. While the enhancement of slow brain rhythms in posterior areas (indicating a vagal contribution) was similar, the increase in power in prefrontal regions and the enhanced connectivity in both theta and high-beta frequencies were observed only during nasal breathing [123]. These findings suggest that nasal breathing is key in breathwork for inducing altered states that may benefit mental health. Nevertheless, a longitudinal study comparing nasal and mouth breathing with proper control would be valuable. Would the long-term psychological effects differ significantly?

Among nasal breathing techniques, one may encounter techniques that focus on breathing through specific nostrils. According to traditional yogic concepts, left nostril breathing (LNB) is believed to activate the PNS, promoting relaxation and a calming effect, whereas right nostril breathing (RNB) is associated with increased SNS activity, leading to heightened psychophysiological arousal [181]. Several early studies have reported differential effects of LNB and RNB on autonomic modulation [182,183,184,185,186]. However, more recent studies, characterized by generally higher methodological quality, have found no significant differences between RNB and LNB in their effects on HRV, BP, or oxygen consumption. Instead, the authors suggest that vagal nerve activity and brain processes related to attentional and volitional breathing may be the primary mechanisms underlying the observed changes [181,187,188,189,190]. Some recent studies have also reported differences in brain activity between RNB and LNB [188,191], but the significance of these changes in terms of their practical relevance remains an open question. The general scarcity of longitudinal studies on RNB and LNB prevents drawing firm conclusions regarding their distinct long-term impacts.

Another widely practiced nostril-based technique is alternate-nostril breathing (ANB), which involves inhaling through one nostril, exhaling through the other, then switching sides and repeating [192]. Two recent systematic reviews have supported the effectiveness of ANB in producing positive psychophysiological effects on human health [148,193]. However, studies directly comparing the long-term effects of ANB with placebo interventions or other breathwork techniques remain limited. One study comparing ANB with a control, paced-breathing condition using the same respiratory rate found similar effects on autonomic modulation in both interventions [192]. Another study, also using a comparable breathing pace (~10 bpm), found that diaphragmatic breathing produced significantly greater increases in HRV parameters than ANB (specifically in RMSSD and HF, *p* < 0.01) [194]. Further well-controlled studies are needed to clarify differences between nostril-based and other breathwork techniques.

### 5.5. Biofeedback and Resonance Frequency

HRVB has been designed to optimize and maximize the efficiency of breathwork by focusing on reaching an individual’s resonance frequency (but it may also serve only as visual feedback of RSA, respiratory rate, or HR) [195]. However, determining the exact resonance frequency remains a controversial issue, particularly regarding its stability over time [196,197,198] and the fact that the phase relationship between breathing and HR appears to deviate from theoretical models in older individuals [199]. Moreover, severe methodological discrepancies persist in HRVB research, with many studies failing to provide sufficient detail or monitor crucial parameters such as breathing rate and ratio. As a result, replicating findings and establishing a “gold standard” for HRVB interventions remains challenging [196].

Nevertheless, this does not diminish the existing evidence supporting HRVB’s effectiveness in influencing both physiology and mental health but rather raises the question of whether HRVB is truly more effective than simple slow-paced breathing without additional equipment [200,201].

Two early reports on this subject [202,203] indicated a slight advantage of HRVB over control conditions based on slow breathing, whereas one study found no such difference [204]. Further work by Laborde et al. [195] has found no psychophysiological differences between a short, five-minute coherent breathing intervention with or without biofeedback, although biofeedback served only as HR visualization. In contrast, the most recent study, which utilized HRVB biofeedback to determine resonance frequency, has shown this intervention to have advantages over coherent breathing in reducing BP, enhancing autonomic regulation, and strengthening brain-cardiovascular interactions. Notably, resonance breathing induced the strongest cardiorespiratory coupling and greater activation in the prefrontal cortex and alpha wave modulation, associated with parasympathetic control [205]. However, the two most recent meta-analyses did not confirm the superiority of biofeedback-assisted resonance breathing for treating anxiety [201], nor did they find generally stronger effects on mental health compared to coherent breathing [206]. More long-term studies directly comparing resonance frequency breathing with placebo controls are needed to definitively determine whether HRVB offers any advantage over simple paced breathing, as current evidence suggests rather similar effects [95].

## 6. Mental Health Disorders

### 6.1. Background

Allostatic load contributes to the development of MD by destabilizing the ANS and affecting the HPA axis. These disturbances have been associated with excessive release of stress hormones and neurotransmitter imbalances, which alter brain function [57,207,208,209,210,211,212]. Under stress, the CNS negatively affects the baroreflex, reducing baseline vagus nerve activity and RSA amplitudes [103]. Impaired autonomic function in anxiety, depression, and post-traumatic stress disorder, as well as during periods of stress, is reflected in reduced HRV [69,213,214,215,216]. Low HRV and higher allostatic load have been associated with greater intolerance to uncertainty and negative thinking, further driving the deterioration of mental health [217,218].

Moreover, there is a strong link between depression and cardiovascular disease, with low RMSSD values (25–35 ms) having emerged as a promising biomarker indicating and predicting the increased risk of both conditions [219]. While pharmacological treatments and psychotherapy have been shown to have a positive effect on depressive disorder symptoms, they have not been shown to improve HRV [220,221]. Depression and anxiety are also bidirectional risk factors for one another, further reducing psychological resilience to social factors [222].

### 6.2. Aiming at Risk Factors

**Figure 3:**
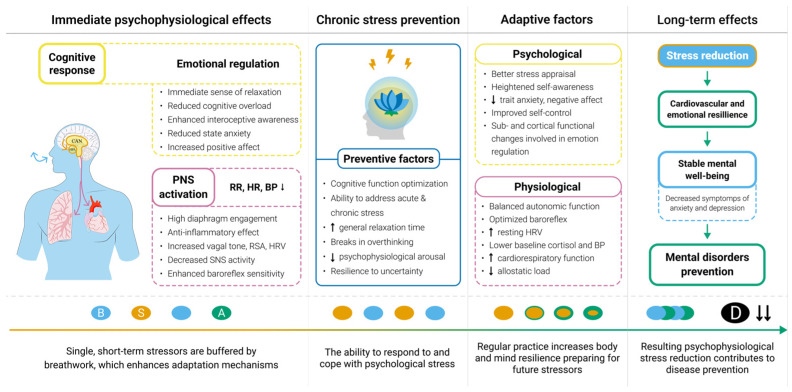
The effects of slow-paced breathwork on mental health. Note: This figure outlines the progression from the immediate psychophysiological effects of slow-paced breathwork to chronic stress prevention and the emergence of adaptive factors, ultimately leading to long-term health benefits. Conscious, slow breathing elicits favorable cognitive and physiological responses, which promote a rapid return to homeostasis when stressors subside. By practicing breathwork regularly, individuals may develop preventive factors against chronic stress, buffering the negative effects of ongoing stressors. Prolonged engagement in breathwork fosters both psychological and physiological resilience, ultimately supporting stress reduction and general resilience, preventing mental disorders. Abbreviations: A (adaptation, green circle), B (breathing technique, blue circle), BP (blood pressure), D (disease progression, black circle), HPA (hypothalamic-pituitary-adrenal axis), HR (heart rate), HRV (heart rate variability), RR (respiratory rate), RSA (respiratory sinus arrhythmia), S (stress, orange circle), SNS (sympathetic nervous system), ↑ (increase), ↓ (decrease). Selected artwork used in this figure (lung, heart, brain, body outline) was adapted from pictures provided by Servier Medical Art (Servier; https://smart.servier.com/), (accessed on 17 March 2025). Licensed under a Creative Commons Attribution 4.0 Unported License.

Emerging evidence suggests that breathwork may be associated with improvements in psychological well-being through its effects on physiological responses, HRV, brain activity, cognitive function, and mood (Figure 3) [13,90]. For instance, the increase in HRV has been associated with enhanced oscillatory activity in brain regions associated with emotional regulation [223]. Therefore, maintaining high HRV levels, even under chronic stress, may help individuals manage uncertainty more effectively, likely due to improved autonomic regulation that enhances resilience to stress [217,223].

Furthermore, breathwork has been conceptualized to address key risk factors of depression and anxiety by enhancing attention and interoceptive awareness and improving self-regulation. By consciously adjusting breath patterns, individuals may gain a greater sense of control over physiological responses to stress, reducing emotional reactivity, interrupting cycles of rumination, and promoting a more adaptive regulation of arousal in challenging situations [139,168].

In an 8-week RCT conducted in China by Ma et al., involving 40 participants, the experimental group performed 20 sessions of 15-min slow (~4 bpm) mindful diaphragmatic breathing, while the control group performed normal breathing. Compared to baseline, statistically significant reductions in cortisol levels and negative affect were observed, along with substantial improvements in sustained attention in the diaphragmatic breathing group [224]. Sustained attention, a crucial cognitive function, is often impaired in individuals with depression and anxiety. Despite the severe impact of attentional deficits on quality of life, they remain largely unaddressed by pharmacological treatments [225,226].

It could be assumed that all breathwork techniques fundamentally rely on the direction and maintenance of sustained focused attention toward changing bodily reactions, thus showing preventive potential [112]. A pilot RCT study conducted in the United States among university students (novice practitioners), comparing the effects of two components of yoga (movement-focused and breath-focused practices), demonstrated reductions in perceived stress and cortisol levels after an 8-week program [227]. However, only the breath-focused group showed improvements in sustained attention. An intriguing finding from the study is the correlation between improved sustained attention and reduced perceived stress, though no such link was found with lowered cortisol levels. This may suggest that physiological responses are not the sole measurable pathway linking the effectiveness of breathing techniques to enhanced mental well-being. Cognitive factors may play an important role in these improvements [227]. The limitation of this study, however, lies in the single-day pre- and post-intervention sampling and the use of a varied set of breathwork exercises, which affect the generalizability of the results [227].

Novaes et al. [153] conducted an RCT in Brazil, investigating the effects of a one-month, thrice-weekly Bhastrika pranayama practice (a combination of slow ANB, fast breathing, and muscle contractions) on functional magnetic resonance imaging outcomes and psychometric measures in healthy individuals [153]. Compared to the active control group (engaged in various cognitive activities), the breathing group exhibited statistically significant changes in brain functional activity and connectivity in regions such as the amygdala, anterior cingulate cortex, anterior insula, and prefrontal cortex. These areas, involved in attention, emotion processing, and self-awareness, were modulated in ways that promoted positive affect while reducing anxiety and negative affect [153]. Notably, the least reductions in negative affect were linked to higher activity in the right amygdala and anterior insula, a pattern characteristic of more anxious individuals [153,228].

It is important to recognize that higher breathing rates may induce neural responses similar to those observed in anxiety and should therefore be used cautiously in patients with MD [112]. In contrast, slow-paced breathwork may reduce anxiety by decreasing anterior insula and amygdala activity, reshaping habitual responses to anticipated stress [82,139]. Increased anterior insula activity is negatively correlated with HRV [136]. Overactivation of this brain region during expected stress has been linked to lower perceived control in anxious individuals [229].

Increased amygdala activity has also been implicated in pain perception, where the relationship between pain and anxiety is bidirectional and mutually reinforcing [230]. A study of 169 individuals with chronic pain from Poland and Spain found that a single session of diaphragmatic breathing significantly reduced perceived pain and anxiety, with stronger effects observed in participants with higher baseline levels [230]. Notably, significant differences between the two nationalities were observed, suggesting that cultural factors influence the consistency and effectiveness of the practice. The authors emphasized that experience with breathwork, including the ability to maintain focus and attentional control, is particularly important, as distractions during practice negatively impact outcomes [230].

Since both anxiety disorders and depression are associated with reduced HRV, and limbic structures mediate this relationship [216,231], breathwork represents a promising approach to addressing this key risk factor [232]. Additionally, breathwork helps individuals manage stress by enhancing body and mind awareness, fostering acceptance of anxiety symptoms, and ultimately leading to better control over stress reactions [201].

Even brief, five-minute sessions of various breathwork techniques have been shown to enhance parasympathetic activation, reduce physiological arousal and perceived anxiety levels, and positively impact brain activity, HRV, and mood [93,117,139,233,234]. These effects may be particularly beneficial for older individuals [234] and those experiencing higher stress levels [235]. Importantly, for effective stress reduction, maintaining regular and consistent practice appears to be more important than extending the duration of individual sessions [20,93], though certain benefits of longer sessions have also been observed [93,233].

### 6.3. The Use of Breathwork for Stress and Mental Health: Systematic Reviews and Meta-Analyses

Recently, the first meta-analysis of RCTs identified breathwork as a promising and effective approach for alleviating stress [19]. Fincham et al. have analyzed data from 12 RCTs involving 785 adults, revealing that breathwork significantly reduces subjective psychological distress (*p* = 0.0009), with small to medium effect sizes observed post-intervention compared to control groups. Two subsequent meta-analyses, including a total of 38 studies, also indicated that breathwork interventions significantly (*p* < 0.0001) improve mental health by alleviating symptoms of anxiety and depression. The effect sizes were small to medium, though the results may have been influenced by variables other than breathwork itself. Despite these findings, the moderate risk of bias across studies, cross-cultural differences, the insufficient number of clinical samples, and the lack of comparisons to non-breathwork controls highlight the need for further research with well-defined participant groups, larger sample sizes, and standardized objective measures [19].

Malviya et al. have reached similar conclusions in their systematic review, both regarding breathwork’s impact on mental health and the methodological limitations found in reviewed studies. Among the 15 included studies, eight examined the effects of breathwork on anxiety and consistently reported statistically significant reductions in symptoms. Depression was also investigated in eight studies; although all observed reductions in depressive symptoms, statistical significance was achieved in only five [236]. It is worth noting that among the 11 RCTs utilizing breathwork, six were conducted in India, four in the United States, and one in Iran. Notably, 75% of the U.S.-based studies had a sample size below 30 participants. Breathwork effectiveness has been demonstrated across all included studies, regardless of participants’ religious or spiritual affiliation. However, the influence of such affiliation cannot be entirely ruled out, as it was not assessed as a variable in any of the studies [236].

Another systematic review conducted by Bentley et al. and including 58 clinical studies has recognized the effectiveness (at a statistical significance of *p* < 0.05) of breathwork in reducing anxiety and/or stress in most (54) of the 72 analyzed interventions, both in healthy and clinical populations, highlighting its potential benefits for young and high-anxiety individuals. In their framework for designing effective breathwork interventions, the authors emphasize the importance of long-term practice, multiple sessions, and initial human-guided training in the initial sessions. They have also demonstrated the low effectiveness of very short (<5 min) practices and those based exclusively on fast breathing, while showing that breathing pace itself is less critical, with combined interventions yielding significant positive outcomes [20].

Regarding HRVB, three meta-analyses have shown significant effects of this technique in improving mental health. Lehrer et al. reviewed 58 papers, indicating that HRVB, as well as coherent breathing, had the most pronounced effects in treating anxiety, depression, and anger, with smaller effects on stress, sleep, and quality of life [206]. The study found small to medium overall effect sizes, which were notably inflated by three outlying studies; when removed, the effect size was smaller but remained significant [206]. Goessl et al., in their analysis of 24 studies involving 484 participants, found that HRVB was associated with substantial reductions in self-reported stress and anxiety. The reported effect sizes for both within-group and between-group (matched against conditions including wait-list controls, standard care, treatment as usual, sham biofeedback, and progressive muscle relaxation) comparisons were large (*p* < 0.001) [237]. Pizzoli et al., based on 14 RCTs, demonstrated that HRVB has a moderate, statistically significant effect in reducing depressive symptoms in adults compared to control and active treatment conditions, with effect sizes comparable to other broadly applied approaches such as cognitive behavioral therapies. The authors observed higher effectiveness in more recent research due to advancements in biofeedback technology and research methodologies [238].

In conclusion, the highest level of evidence indicates that breathwork may serve as a complementary intervention for mental health issues, while emphasizing the need for further research in clinical populations.

### 6.4. Clinical Context

Recently, Kavitha et al. conducted an RCT in India involving 140 patients (112 participants completed the study) with anxiety, investigating the effects of incorporating a 20-min slow pranayama session followed by a 10-min relaxation pose (savasana) into a standard psychiatric care routine [232]. After eight weeks of regular practice, the majority of cardiovascular parameters showed statistically significant (*p* < 0.05) improvements in the pranayama group compared to the control group (routine psychiatric care). These improvements included decreases in resting HR, BP, and LF power, as well as increases in resting HRV parameters such as RMSSD, SDNN, and HF power, indicating better sympathovagal balance and autonomic reactivity with greater PNS activity [232].

Given the limited effectiveness of standard depression treatments in improving HRV, Caldwell et al. examined the impact of adding HRVB to psychotherapy. After six weeks, the combined intervention group showed a greater reduction in depressive symptoms and a significant increase in HRV compared to control groups, suggesting HRVB as an effective supplementary intervention [221]. In another study, Blades et al. compared the Wim Hof Method (WHM), which is based on fast breathwork, with an active control intervention incorporating slow-paced breathing in 84 women with severe depressive symptoms and at risk of clinical depression [239]. After a three-week intervention and at a three-month follow-up, both groups showed reductions in depression, anxiety, and perceived stress, as well as lower cortisol reactivity to acute stress. However, while the WHM group exhibited significantly less daily rumination after stress exposure, participants reported lower satisfaction with the high-frequency breathing technique. This dissatisfaction may have stemmed from the incorporation of cold showers in the WHM protocol [239]. It is possible that fast breathwork, which initially induces higher SNS activity, may help prepare individuals for real-life stress by intentionally triggering a controlled stress response [133]. However, such techniques should be practiced in a safe environment, as they carry the risk of adverse effects. Sharma et al., in their RCT, examined the effectiveness of breathwork in 25 patients with major depressive disorder who had not responded to antidepressant treatment. The application of the Sudarshan Kriya Yoga (SKY) program, a combination of slow and fast breathing techniques, over eight weeks resulted in statistically significant improvements in symptoms of depression and anxiety compared to a wait-list control group [240]. However, it is noteworthy that, while the aforementioned findings are promising, they are based on a relatively small sample size (n = 25), requiring further replication in larger trials.

Lastly, anxiety and panic disorder have been interrelated with abnormal respiratory patterns and DB [201,241,242]. The positive feedback loop between anxiety, increased sympathetic activity, and thoracic dominant breathing may worsen ventilation and reduce oxygen availability, potentially leading to cognitive impairment and heightened panic and anxiety symptoms [168,201]. Furthermore, numerous studies have highlighted a possible association between respiratory and olfactory dysfunctions, particularly those related to nasal obstruction and comorbid psychiatric symptoms [60,165,243]. Therefore, addressing DB appears to be highly beneficial in managing MD.

Leyro et al. conducted a systematic review and meta-analysis of 40 RCTs exploring the potential of respiratory interventions targeting DB for anxiety treatment [201]. They reported medium to large improvements in anxiety symptoms compared to controls, particularly inactive ones, with notable effects on dimensional anxiety and panic symptoms. By correcting respiratory dysfunctions, breathwork techniques were found to be effective in alleviating somatic symptoms and reducing physiological arousal [201]. However, the effects were smaller when compared to active controls, and most of the analyzed studies had a high risk of bias, underscoring the need for further research to refine and standardize these interventions. Additionally, the findings highlight the potential of breathwork to enhance anxiety sensitivity and reduce intolerance to uncertainty by fostering a greater sense of control. This suggests that breathwork could possibly serve as a promising approach not only for anxiety management but also for addressing broader comorbid symptoms in both clinical and general populations [201]. One RCT examining the use of a 10-day breathing exercise program in older hospitalized patients with acute COPD exacerbation found that practicing breathwork significantly improved dyspnea, anxiety, and depression compared to the control group [244].

Therefore, the incorporation of breathwork into treatment may be particularly effective for individuals with pre-existing respiratory conditions, who are also more susceptible to co-occurring DB and MD, as exemplified by individuals with asthma [165,168,201,245,246].

## 7. Conclusions and Future Perspectives

In today’s fast-paced world, breathwork has emerged as a simple and valuable tool for enhancing psychological resilience. Growing evidence has recognized its effectiveness as a safe, non-pharmacological approach for managing stress and improving mental health. Based on its strong psychophysiological foundations, breathwork has the potential to effectively target symptoms of anxiety and depression, offering a viable adjunctive intervention that may be seamlessly integrated with standard therapeutic treatments for various mental disorders [160,165,236].

However, certain precautions must be considered, particularly with fast breathing techniques. While controlled hyperventilation has the potential to induce altered states of consciousness that may aid emotional processing, it also carries risks, especially for vulnerable clinical populations. Under controlled therapeutic conditions—and balanced with slow-paced techniques—fast breathwork might help individuals gain better control over their autonomic responses and help reduce stress in critical situations [20,135,247]. Their careful application in supportive settings could be beneficial, particularly for treatment-resistant anxiety and depression [247].

Looking broadly at breathwork, the associated health benefits appear to be extensive, regardless of the specific technique. Based on the latest findings and in line with some authors’ claims, we argue that its long-term effectiveness depends more on volitional breathing regulation in a structured, regularly paced manner than on the often minimal theoretical and methodological differences between techniques [20,133]. The effectiveness of most breathing techniques has been associated with their positive modulatory effects on the PNS, on neurophysiological changes related to stress appraisal, and on the enhancement of attention. While various techniques may yield different short-term psychophysiological effects, their distinctive long-term significance diminishes over time. It is systematic, consistent practice that enables individuals to recognize and interrupt habitual, maladaptive thought patterns and increase overall stress resilience. However, the power of breathwork’s short-term effects should not be underestimated. The ability to regulate acute stress responses situationally may be crucial for preventing chronic stress and reducing risk factors associated with declining mental well-being.

Despite its promise, current research on breathwork is not without limitations, including methodological inconsistencies, small sample sizes, and inadequate control conditions, which make it difficult to draw definitive conclusions. Moreover, some claims may reflect a reliance on traditional practices rather than rigorous scientific validation. Future investigation should address these gaps by incorporating diverse samples and active control groups while exploring additional variables such as participant expectations, individual characteristics, and socio-cultural influences.

The broader implementation of breathwork into daily life holds great promise from both economic and practical perspectives. Its inherent simplicity allows effective practice without the need for expensive equipment or specialized training. While HRVB devices may enhance the practice, evidence suggests that simple, slow-paced techniques can offer comparable benefits. Further comparative studies are needed to evaluate breathwork’s efficacy relative to conventional therapies and other non-pharmacological treatments, such as mindfulness and cognitive-behavioral therapy. Additionally, research should explore potential synergies when these interventions are combined.

## Data Availability

Not applicable.
